# SCAV‐3 affects apoptotic cell degradation in *Caenorhabditis elegans*

**DOI:** 10.1002/2211-5463.13599

**Published:** 2023-04-02

**Authors:** Aiying Ma, Qi Feng, Peiyao Li, Lei Yuan, Hui Xiao

**Affiliations:** ^1^ College of Life Sciences Shaanxi Normal University Xi'an China; ^2^ Biological Science and Engineering College North Minzu University Yinchuan China; ^3^ Ningxia Key Laboratory for the Development and Application of Microbial Resources in Extreme Environments Yinchuan China

**Keywords:** apoptosis, apoptotic cell clearance, *Caenorhabditis elegans*, lysosomes, phagolysosomes, scavenger receptor

## Abstract

As the final step in apoptosis, apoptotic cells (ACs) are swiftly removed by specialized phagocytes, such as macrophages, or nonprofessional phagocytes, such as epidermal cells. Genetic studies of model organisms such as *Caenorhabditis elegans* have helped to elucidate the mechanisms of AC clearance and the underlying causes of disorders related to the dysregulation of these pathways. *C. elegans* possesses six class B scavenger receptor homologs, but whether they affect apoptosis is unknown. Here, we show that only the loss of function of *scav‐3*, the *C. elegans* homolog of human lysosomal integral membrane protein‐2, resulted in a considerable accumulation of cell corpses, which was caused by a failure in degradation rather than engulfment. SCAV‐3 was found to be widely distributed and localized in lysosomes to maintain the integrity of the lysosomal membrane. Further study revealed that loss of *scav‐3* had no effect on phagosome maturation or the recruitment of lysosomes to phagosomes carrying cell corpses. Moreover, we discovered that the hydrolytic enzymes contained in the lysosomes were reduced in phagosomes in *scav‐3* mutants. Thus, hydrolases may leak from the damaged lysosome during phagolysosome formation due to the loss of *scav‐3* function, which reduces lysosome digestion activity and thus directly contributes to the elimination of ACs.

## Graphical abstract

SCAV‐3 is widely distributed and localized in lysosomes in *Caenorhabditis elegans* to maintain the integrity of the lysosomal membrane. Loss of function of SCAV‐3 causes the accumulation of apoptotic cells (ACs). We propose that hydrolases may leak from the damaged lysosome in the process of phagolysosome formation in *scav‐3* mutants, which leads to the accumulation of ACs.

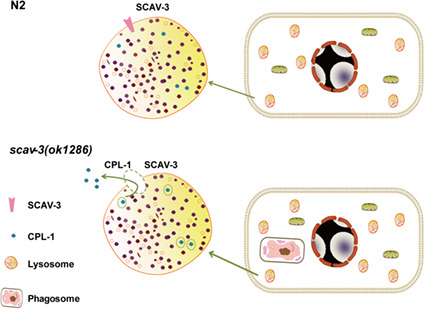

**Abbreviations**
ACs: apoptotic cells
AO: acridine orange
LIMP‐2: lysosomal integral membrane protein‐2
PS: phosphatidylserine
SRB receptors: class B scavenger receptors

As the final step in apoptosis, apoptotic cells (ACs) are swiftly removed by specialized phagocytes such as macrophages or nonprofessional phagocytes such as epidermal cells [1]. The clearance of ACs (efferocytosis) is essential for maintaining tissue homeostasis [2]. In contrast, defective efferocytosis results in the release of harmful contents from ACs, which leads to chronic severe autoimmune diseases and neurodegenerative diseases [3].

Genetic studies of model organisms such as *Caenorhabditis elegans* have helped to understand AC clearance mechanisms and the underlying causes of disorders related to the dysregulation of these pathways. In *C. elegans*, two parallel redundant regulatory pathways control the process of engulfment of ACs. In the *ced‐1/6/7* pathway [4, 5, 6], the phagocytic receptor CED‐1 recognizes ‘eat‐me’ signal phosphatidylserine (PS) [7, 8, 9]. In the *ced‐2/5/12* pathway [10, 11, 12, 13], several receptors including the phosphatidylserine receptor PSR‐1 [14], MOM‐5, and integrin INA‐1 [15] are proposed to recognize and bind to the PS.

It is reasonable to propose that the engulfment of ACs in *C. elegans* is mediated by additional receptor‐based recognition systems. CD36 and SR‐BI, two B‐class scavenger receptors, have played crucial roles in AC clearance in mammals, but the precise molecular mechanism is unclear [16, 17, 18]. SCAV‐1–6 comprises the six class B scavenger receptor homologs found in *C. elegans* (SRB). However, whether SCAV‐1–6 affect apoptosis in *C. elegans* is unknown.

In the present study, we found that only loss of function of SCAV‐3 causes the accumulation of ACs in *C. elegans*. We show that *scav‐3* acts specifically in phagocytes for cell corpse clearance. In addition, we found that the persistent cell corpses in *scav‐3* mutants were internalized but failed to be degraded promptly. Consistent with previous studies, we found that SCAV‐3 is widely distributed and localized on lysosomes to maintain the integrity of the lysosomal membrane, and loss of *scav‐3* causes damage to lysosome membranes. Our further analysis found that loss of *scav‐3* did not affect the phagosome maturation process or the recruitment of lysosomes to the cell corpse‐containing phagosome. Furthermore, we discovered that lysosomal hydrolytic enzymes could enter phagosomes, but were reduced in phagosomes in *scav‐3* mutants. We propose that hydrolases may leak from damaged lysosomes during the formation of phagolysosomes in *scav‐3* mutants, leading to the accumulation of ACs. Our findings provide direct evidence for the significance of lysosomal membrane integrity in the removal of ACs during programmed cell death.

## Materials and methods

### *C. elegans* strains and genetics

The Bristol strain N2 was used as the wild‐type. Linkage groups listed other mutant alleles used in this study: LG I: CB3203 [*ced‐1(e1735)*], LG II: *vps‐18(tm1125)*, LG III: RB1127 [*scav‐3(ok1286)*] and RB973 [*scav‐2(ok877)*], and LG X: RB1409 [*scav‐5(ok1606)*]. All mutants were outcrossed at least four times with N2. Integrated arrays used in this study are as follows: *smIs34(P*_*ced‐1*_ *ced‐1::gfp)*, *qxIs66(P*_*ced‐1*_ *gfp::rab‐7)*, *qxIs257(P*_*ced‐1*_ *nuc‐1::mCherry)*, *qxIs352(P*_*ced‐1*_ *laat‐1::mCherry)*, *qxIs354(P*_*ced‐1*_ *laat‐1::gfp)*, *qxIs60(P*_*ced‐1*_ *lmp‐1::gfp)*, *qxIs58(P*_*ced‐1*_ *lmp‐1::mCherry)*, and *yqEx620(P*_*cpl‐1*_ *cpl‐1::mChOint)*. Animals carrying stably integrated arrays were outcrossed with the N2 strain four times. *C. elegans* cultures and genetic crosses were performed essentially according to standard procedures [19].

### Plasmid construction

To construct the plasmid for RNAi, the genomic sequence of the *scav‐1* or *scav‐6* was amplified by PCR and inserted between the *BglII* and *HindIII* sites of the vector *pPD129.36*, and genomic sequence of *scav‐4* was amplified by PCR and inserted between the *XhoI* and *HindIII* sites of the vector *pPD129.36*. To make the construct of *P*_*scav‐3*_ *scav‐3::gfp*, a 5.3‐kb fragment containing the genomic sequence of the gene and 2.1 kb of its promoter region was inserted between the *HindIII* and *BamHI* sites of the vector *pPD95.77*. To make *P*_*ced‐1*_ *scav‐3* and *P*_*egl‐1*_ *scav‐3* constructs, the full‐length *scav‐3* cDNA was cloned into the *P*_*ced‐1*_ vector via its *SalI* and *BamHI* sites or into the *P*_*egl‐1*_ vector through its *BamHI* and *XmaI* sites.

### RNAi experiments

RNAi experiments were performed by using bacterial feeding assays as described previously [20]. In most cases, L4‐stage animals were transferred to plates seeded with bacteria expressing either control double‐stranded RNA (dsRNA; *L4440* empty vector; Control RNAi) or dsRNA of genes of interest. Germ and embryo cell corpses were observed at different time points.

### Quantification of cell corpses

Cell corpses were scored by using Nomarski optics. For embryonic cell corpses, only those in the head regions of embryos at the comma, 1.5‐fold, twofold, threefold, and fourfold stages were scored. At least 15 embryos at each embryonic stage were scored, and the average numbers of cell corpses were shown. Germ cell corpses in the germline meiotic region of one gonad arm were scored 12, 24, 36, 48, and 60 h after the L4 larval stage. Data derived from different genetic backgrounds were compared using unpaired *t*‐tests.

### Acridine orange staining of cell corpses

To stain the germ cell corpses with Acridine orange (AO), adult worms were incubated in M9 medium containing AO (100 mg·mL^−1^) and OP50 bacteria in the dark for 2 h at room temperature. Worms were then transferred to nematode growth medium plates for recovery for 1 h to decrease background gut fluorescence. The stained worms were examined under fluorescence microscopy to score the AO‐positive germ cell corpses from > 150 germ cell corpses of each examined strain.

### Four‐dimensional analysis of cell corpse duration

Time‐lapse imaging was performed under 100× objectives at 20 °C. To record the duration of embryonic cell corpses, early embryos (2‐cell stage) were put in egg salt buffer (118 mm NaCl and 48 mm KCl) and mounted on slides with agar pads. The slides were sealed with beeswax and petroleum jelly (1 : 1). Images in 20 Z‐serial sections (1 μm/section) were captured every minute for 400 min by using a Zeiss Axioimager M2 (Zeiss, Imager M2, Oberkochen, Germany) coupled with an AxioCam monochrome digital camera.

## Results

### Loss of function of *scav‐3* caused the accumulation of ACs in *C. elegans*

**Fig. 1:**
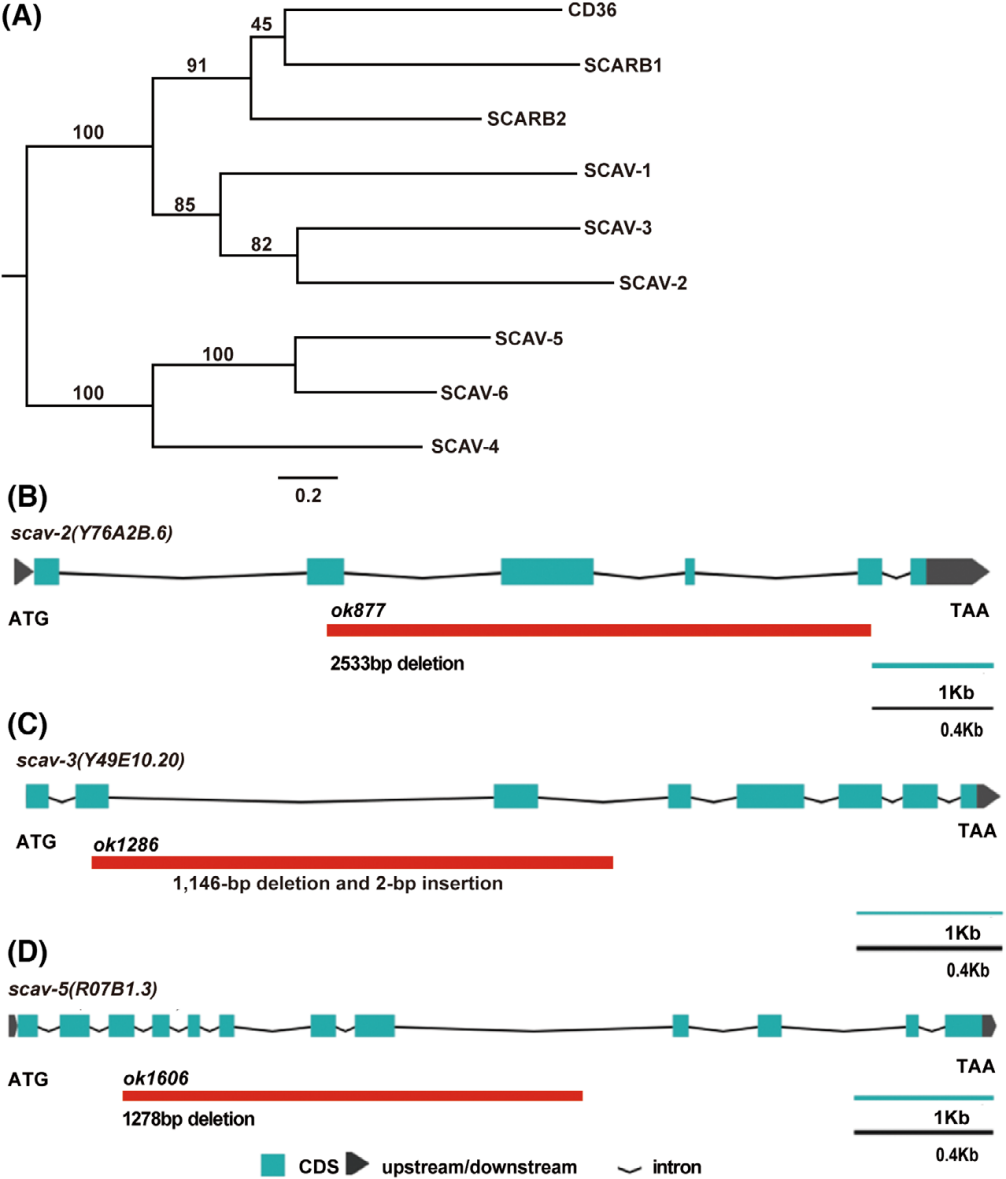
Phylogenetic analysis of SCAV receptors and schematic representation of *scav‐2*/*3*/*5* deletion mutants. (A) The phylogenetic analysis comparing the *Caenorhabditis elegans* and human SCAV receptors. (B–D) Schematic representation of *scav‐2(ok877)*, *scav‐3(ok1286)*, and *scav‐5(ok1606)* deletion mutants. Solid boxes indicate exons and waved lines indicate introns. The fragment below each gene indicates the region and size of deletion mutation of each *scav* gene. The scale bar represents 1000 base pairs for exons and 400 base pairs for introns, respectively.

**Fig. 2:**
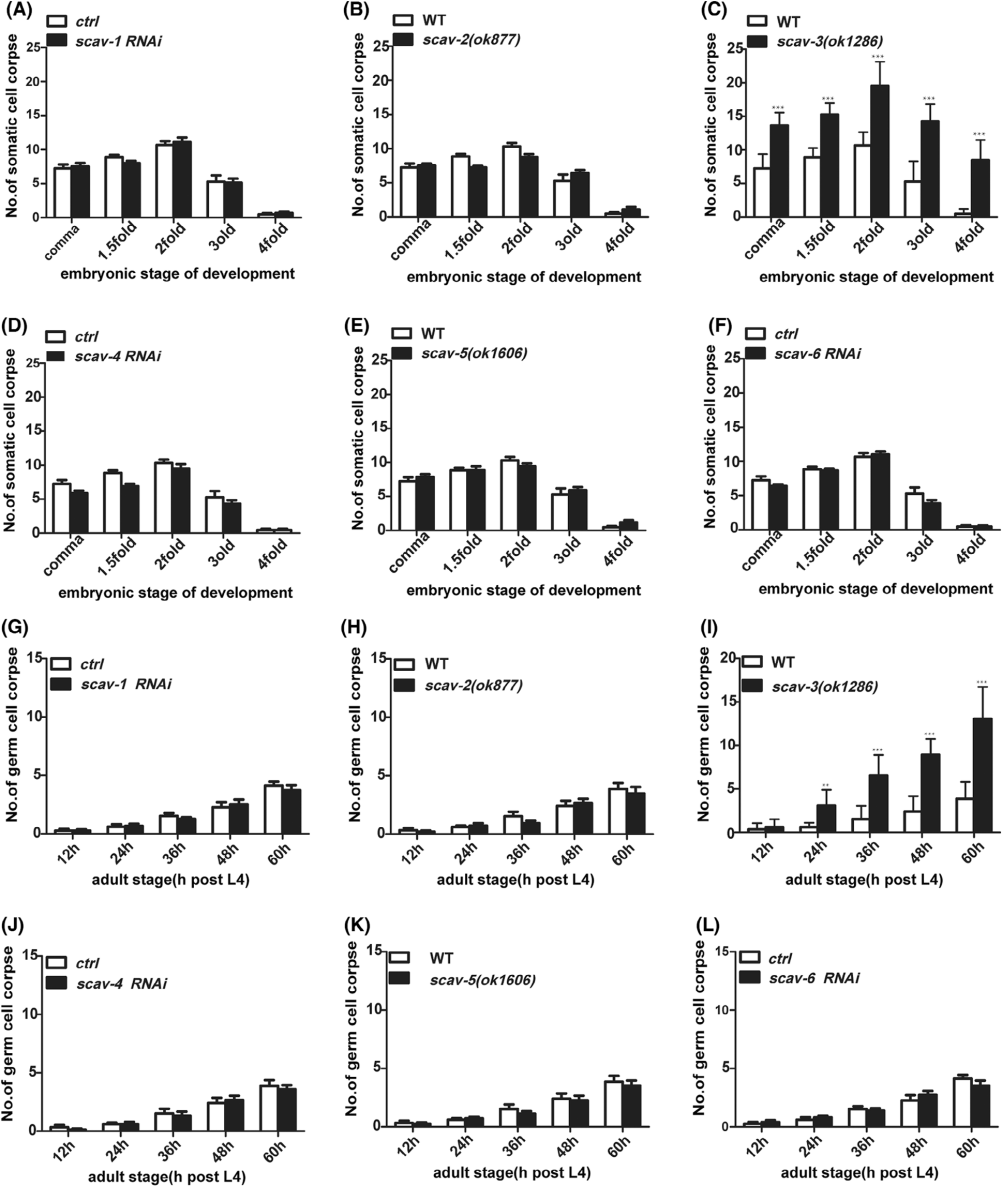
Loss‐of‐function mutation of *scav‐3* caused accumulation of ACs in *Caenorhabditis elegans*. The profile of embryonic cell death in (A) *scav‐1* RNAi‐treated, (B) *scav‐2(ok877)* mutants, (C) *scav‐3(ok1286)* mutants, (D) *scav‐4* RNAi‐treated, (E) *scav‐5(ok1606)* mutants, and (F) *scav‐6* RNAi‐treated. Cell corpses of each animal were scored in ≥ 15 animals at every time point as indicated. Quantification of germ cell corpses in (G) *scav‐1* RNAi‐treated, (H) *scav‐2(ok877)* mutants, (I) *scav‐3(ok1286)* mutants, (J) *scav‐4* RNAi‐treated, (K) *scav‐5(ok1606)* mutants, and (L) *scav‐6* RNAi‐treated. Cell corpses in one gonad arm of each animal were scored in ≥ 15 animals at every time point as indicated. Error bars represent SEM. Comparisons were performed with an unpaired *t*‐test. ***P < 0.001.

The SCAV family in *C. elegans* is highly evolutionarily conserved with the human CD36 family (Fig. 1A). To determine whether *scav‐1*, *scav‐2*, *scav‐3*, *scav‐4*, *scav‐5*, and *scav‐6* are involved in apoptosis in *C. elegans*, we used RNAi interference to knock down *scav‐1*, *scav‐2*, and *scav‐4* to count the number of cell corpses. The number of ACs in *scav‐2(ok877)*, *scav‐3(ok1286)* [21], and *scav‐5(ok1606)* deletion mutants (Fig. 1B–D) was also compared to that of wild‐type animals N2. We found that only *scav‐3(ok1286)* mutants displayed a significant increase in ACs at various stages of embryonic development (Fig. 2A–F). We also observed that germ cell corpses accumulated significantly in an age‐dependent manner in *scav‐3(ok1286)* animals (Fig. 2G–L). In addition, we found that *scav‐3* RNAi‐treated worms had a significantly higher apoptotic phenotype in embryos and germ cells when compared to controls (Fig. S1A,B). The *scav‐3(ok1286)* contains a 1146‐bp deletion and a 2‐bp insertion that removes most of the second and third exons and generates an early stop codon, resulting in a truncated protein containing only the first 118 amino acids, representing a null mutation of the *scav‐3* gene (Fig. 1) [21]. While the *scav‐3* mutant was able to produce healthy fertilized oocytes, we were unable to detect any defects in the mutant's growth while keeping it in culture. These results demonstrate that loss of *scav‐3* function caused apoptotic program abnormalities.

### SCAV‐3 affects cell corpse clearance in *C. elegans*

**Fig. 3:**
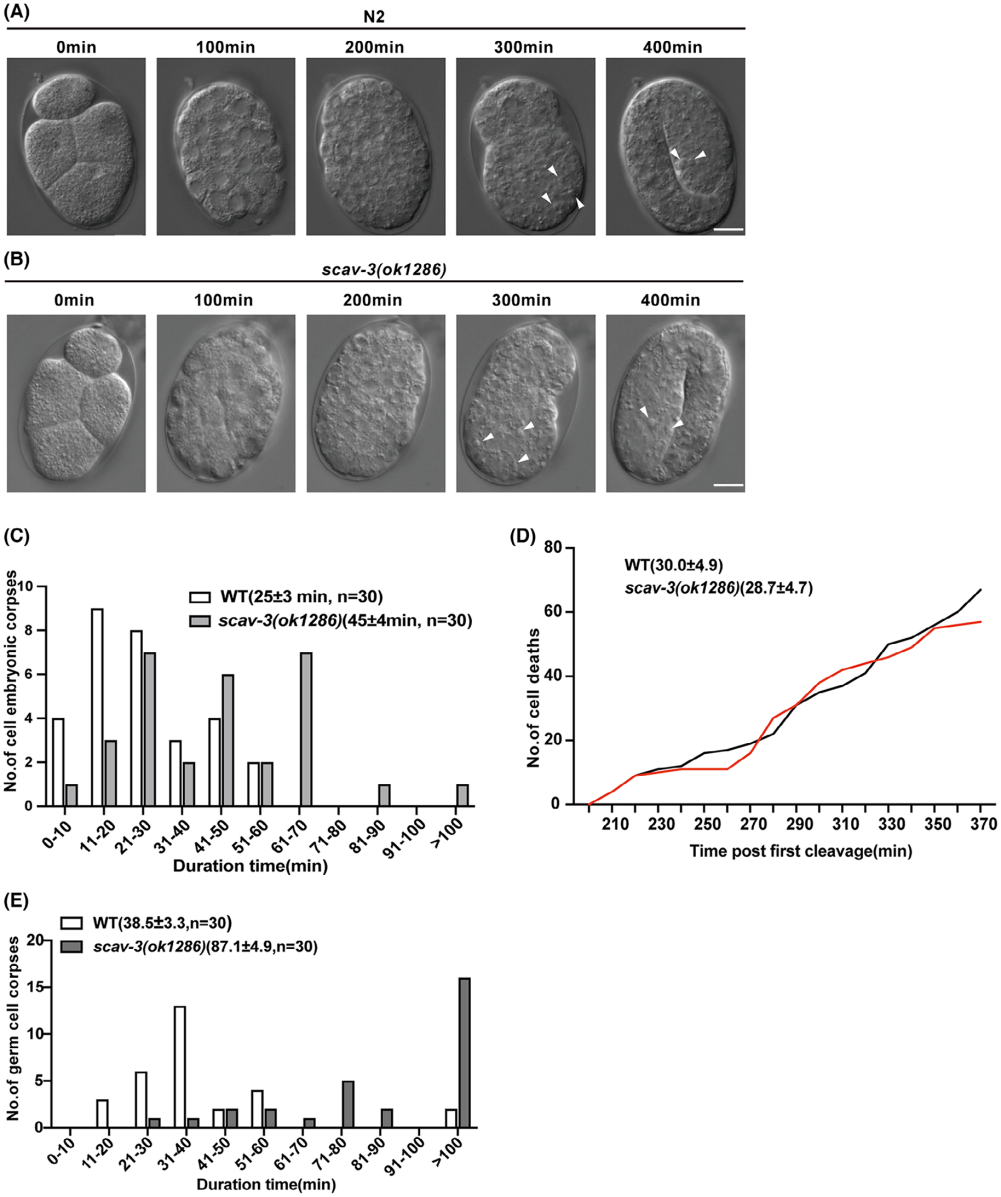
*Caenorhabditis elegans scav‐3* affects cell corpse clearance. Time‐lapse recording of the cell corpses of both (A) wild‐type and (B) *scav‐3(ok1286)* animals. Bars, 10 μm. (C) Four‐dimensional microscopy analysis of embryonic cell corpse duration in N2 and *scav‐3(ok1286)* animals. Thirty embryonic cell corpses were analyzed for each genotype. The numbers in parentheses indicate the average duration of cell corpses (mean ± SEM). (D) The embryos selected in the experiment started with the first four cells. ACs began to appear in embryos at 200 min, marked by the appearance of ‘button‐shaped’ and wrinkled ACs on the embryo surface. The number of ACs produced was counted every 10 min, and the broken line map was made. The mean number of total cell deaths (mean ± SD) is shown in parentheses. The y axis indicates the number of cell deaths at each time point, *P* > 0.05. (E) Four‐dimensional microscopy analysis of durations of persistence of germ cell corpses in N2 and *scav‐3(ok1286)* animals. Thirty germ cell corpses were analyzed for each genotype. The numbers in parentheses indicate the average duration of cell corpses (mean ± SEM).

The increase of both embryonic and germ cell corpses in *scav‐3(ok1286)* could be caused by either excessive apoptosis or cell corpse clearance deficiencies. To distinguish between these two possibilities, we first used the promoter of *ced‐1*, which is only expressed in engulfing cells, to drive the expression of *scav‐3* (P_*ced‐1*_ *scav‐3*) and discovered that it fully rescued the defects in cell corpse clearance in *scav‐3(ok1286)* embryos (Table 1) as well as in adult germ lines (Table 2). In comparison, we also used the promoter of *egl‐1* [22, 23, 24], which was exclusively expressed in dying cells, to drive the expression of *scav‐3* (*P*_*egl‐1*_ *scav‐3*) but found it did not rescue the cell corpse clearance defects in *scav‐3(ok1286)* embryos (Table 1). We next carried out time‐lapse recording analysis to measure the duration of persistence of both embryonic (Fig. 3A,B) and germ cell corpses. In wild‐type animals, the average duration of embryonic cell corpses was 25 ± 3 min, whereas 60% of cell corpses persisted more than 30 min and some exceeded 100 min in *scav‐3(ok1286)* animals (Fig. 3C). In germ lines, the average duration of germ cell corpses was 51.7 ± 5.8 min, whereas we seldom observed germ cell corpses persisting 60 min in wild‐type animals. In *scav‐3(ok1286)* mutant, the average persistence time of germ cell corpses significantly increased, with ≥ 90% of examined cell corpses persisted over 100 min (Fig. 3E). Moreover, there were no significant differences between wild‐type and *scav‐3(ok1286)* in the number of cell death events from the first cell division to 400 min during embryogenesis (Fig. 3D). We then monitored the uptake and degradation of ssGFP by coelomocytes in a time course manner. At 20 h after heat shock, ssGFP in the body cavity and coelomocytes had vanished in the wild‐type. However, ssGFP was observed both in the body cavity and in coelomocytes even 36 h after heat shock in *scav‐3* mutant animals, indicating that endosomal/lysosomal protein degradation was strongly impeded in the mutant coelomocytes (Fig. S2A,B). Together, these findings demonstrate that SCAV‐3 activity in engulfing cells is essential for cell corpse clearance, and the increase of cell corpses in *scav‐3(ok1286)* animals is caused by defects in the removal of cell corpses.

**Table 1:** Expression of *scav‐3* cDNA in engulfing but not dying cells rescued a *scav‐3* phenotype in embryo of *scav‐3(ok1286)*.

Genotype	Transgene	No. corpses of embryo					
		Comma	1.5‐fold	2‐fold	2.5‐fold	3‐fold	4‐fold
N2	–^a^	8.6 ± 1.6	8.9 ± 1.4	14 ± 1.9	7.7 ± 1.4	4.5 ± 3.0	0.5 ± 0.7
*scav‐3(ok1286)*	–	11.5 ± 2.2**^,^ ^b^	15.3 ± 1.7***	19.3 ± 3.6***	22 ± 3.6***	14.2 ± 2.6***	8.5 ± 3.0***
*scav‐3(ok1286)*	*Pced‐1 scav‐3 line1* ^c^	10.2 ± 1.4	10.3 ± 1.8^###^	15.2 ± 2.8^###^	9.8 ± 2.1^###^	6.1 ± 1.4^###^	0.9 ± 1.0^###^
*scav‐3(ok1286)*	*Pced‐1 scav‐3 line2*	8.9 ± 1.4^#^	10.9 ± 2.0^###^	15 ± 1.8^###^	8.3 ± 2.3^###^	4.9 ± 2.2^###^	0.9 ± 0.7^###^
*scav‐3(ok1286)*	*Pced‐1 scav‐3 line3*	8.9 ± 1.4^#^	10.9 ± 1.9^###^	15 ± 1.8^###^	8.3 ± 2.3^###^	5 ± 2.6^###^	0.9 ± 0.7^###^
*scav‐3(ok1286)*	*Pegl‐1 scav‐3 line1*	11.9 ± 2.4***	13.6 ± 2.4***	19.8 ± 1.7***	22.6 ± 2.7***	12 ± 1.7***	4.1 ± 2.0***
*scav‐3(ok1286)*	*Pegl‐1 scav‐3 line2*	12.5 ± 2.5***	13.1 ± 2.6***	19.7 ± 2.2***	21.8 ± 3.1***	10.1 ± 2.2***	6.4 ± 1.5***
*scav‐3(ok1286)*	*Pegl‐1 scav‐3 line3*	11.7 ± 2.4***	13.7 ± 1.9***	19.4 ± 2.7***	22.6 ± 3.2***	11.5 ± 2.6***	6.8 ± 2.3***

^a^ For each transgene construct, three independent lines were assayed, −, no transgene.

^b^ Compared with N2 **P < 0.01, ***P < 0.001; compared with *scav‐3(ok1286)* ^#^ *P* < 0.05, ^###^ *P* < 0.001.

^c^ Data are presented as mean ± standard deviation. Cell corpses of each animal were scored in ≥ 15 animals at every time point as indicated.

**Table 2:** Expression of *scav‐3* cDNA in engulfing but not dying cells rescued a *scav‐3* phenotype in germline of *scav‐3(ok1286)*.

Genotype	Transgene^a^	No. corpses of germ line (after L4)				
		12 h	24 h	36 h	48 h	60 h
*N2*	–^a^	0.3 ± 0.7	0.6 ± 0.5	1.5 ± 1.5	2.4 ± 1.7	3.9 ± 1.9
*scav‐3(ok1286)*	–	0.6 ± 0.9^b^	3.1 ± 1.8**	6.5 ± 2.3***	8.9 ± 1.8***	13.1 ± 3.7***
*scav‐3(ok1286)*	*P*_*ced‐1*_ *scav‐3 line1* ^c^	0.1 ± 0.4	1.7 ± 1.6	2.2 ± 2.4^###^	3.2 ± 3.0^###^	4.1 ± 2.9^###^
*scav‐3(ok1286)*	*P*_*ced‐1*_ *scav‐3 line2*	0.1 ± 0.4	1 ± 1.3	3.5 ± 2.6^##^	5.1 ± 3.4^###^	4.1 ± 3.4^###^
*scav‐3(ok1286)*	*P*_*ced‐1*_ *scav‐3 line3*	0.1 ± 0.4	0.6 ± 1.0^###^	1.5 ± 1.2^###^	1.8 ± 1.6^###^	4.4 ± 1.8^###^

^a^ For each transgene construct, three independent lines were assayed, −, no transgene.

^b^ Compared with N2 **P < 0.01, ***P < 0.001. Compared with *scav‐3(ok1286)* ^##^ *P* < 0.01, ^###^ *P* < 0.001.

^c^ Data are presented as mean ± standard deviation. Cell corpses of each animal were scored in ≥ 15 animals at every time point as indicated.

### SCAV‐3 is widely distributed and localizes to lysosomes

**Fig. 4:**
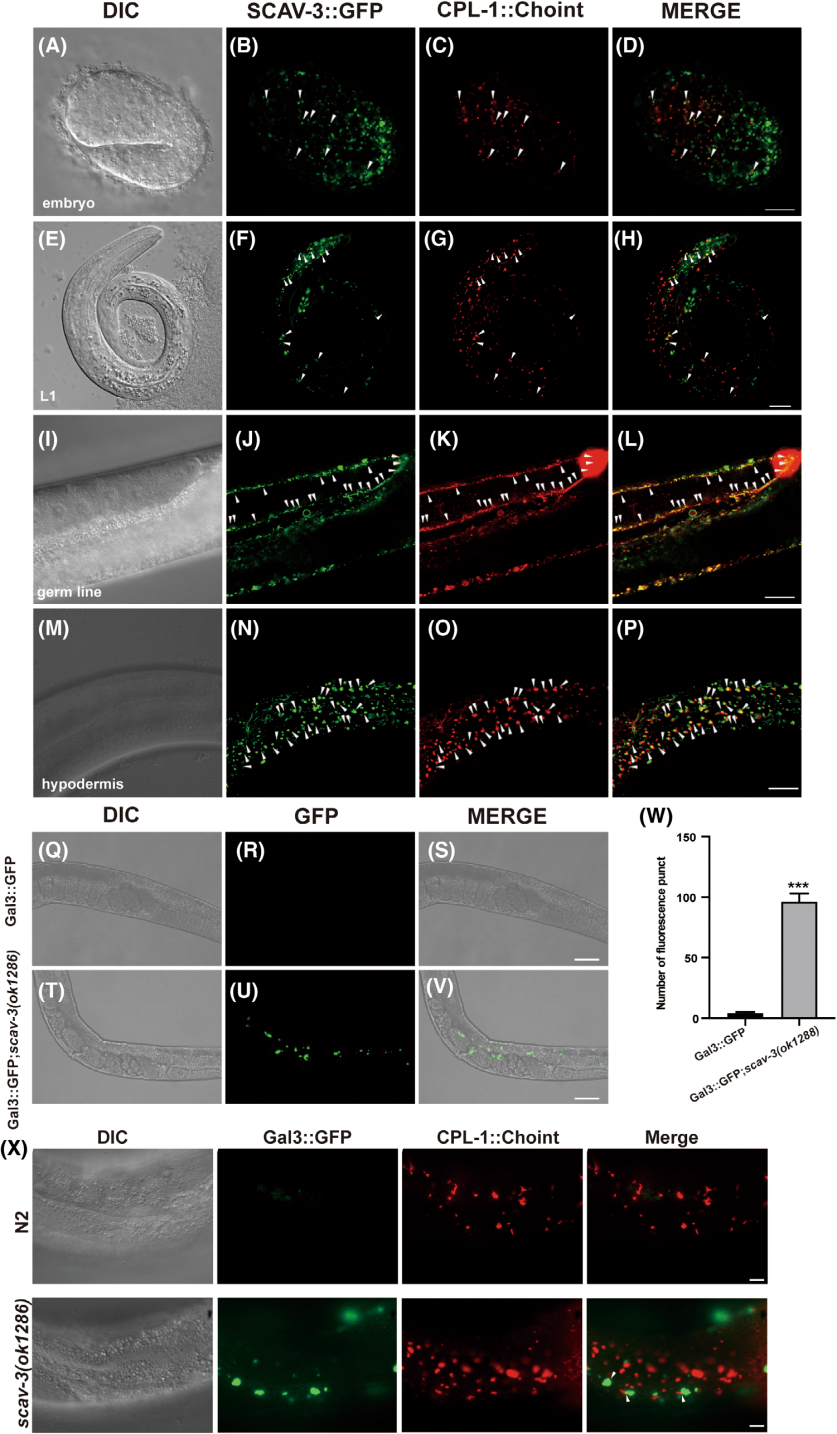
Damaged lysosomes accumulated in *scav‐3* mutants. (A–P) Expression and subcellular localization of SCAV‐3::GFP driven by the *scav‐3* promoter. Images of DIC, SCAV‐3::GFP, and CPL‐1::mChOint (driven by the *cpl‐1* promoter) and merged SCAV‐3::GFP with CPL‐1::mChOint are shown for a 1.5‐fold embryo, an L1 larvae, the germ line, and the hypodermis. Bars, 10 μm. Arrowheads indicate some of the foci with colocalization of SCAV‐3::GFP and CPL‐1::mChOint. The fluorescent images of the hypodermis in wild‐type (WT; Q–S) and *scav‐3(ok1286)* (T–V) adults expressing Gal3::GFP. Bars, 50 μm. (W) The average number of positives for GFP::Gal3 (damaged) was quantified in wild‐type and *scav‐3(ok1286)* expressing Gal3::GFP. At least 15 animals were scored in each strain (*n* = 3, mean ± SEM). (X) The fluorescent images of the hypodermis in wild‐type and *scav‐3(ok1286)* adults expressing Gal3::GFP and CPL‐1::mChOint. Colocalization indicated by white arrows; Bars, 10 μm. Data were compared with the unpaired *t*‐test. ***P < 0.001.

The open reading frame *Y49E10.20* found on linkage group III and encoding the 534 amino acid SCAV‐3 protein in *C. elegans*. Previous studies have found that SCAV‐3 can maintain the integrity of the lysosomal membrane and that aberrant SCAV‐3 function can affect the life span of *C. elegans* [17]. We generated a SCAV‐3::GFP reporter driven by the *scav‐3* promoter and found that SCAV‐3::GFP is widely expressed during embryonic (Fig. 4A–D), larval (Fig. 4E–H), and adult stages in various tissues (Fig. 4I–L,M–P). SCAV‐3::GFP‐labeled puncta structures were positive for CPL‐1::mChOint at various stages [25], especially the colocalization rate in gonads and epidermal tissues nearly 90%, indicating that SCAV‐3 localizes to lysosomes. Gal3 is a β‐galactoside‐binding protein and is used to detect endosomal or lysosomal damage [26]. Consistent with the previous study, we found that GFP::Gal3 was accumulated in *scav‐3(ok1286)* mutants (Fig. 4Q–V). The average number of Gal3::GFP in *scav‐3(ok1286)* is significantly higher than in N2 (*P* < 0.001) (Fig. 4W). We monitored GAL3::GFP together with the lysosomal marker CPL‐1::mChOint and observed GAL3::GFP partially colocalized with CPL‐1::mChOint (Fig. 4X). We also discovered that the colocalization of GAL3 and CPL‐1 in *scav‐3* mutant is weaker than in the wild‐type, which may be due to the leakage of CPL‐1 from the damaged lysosomes of the *scav‐3* mutant. These results suggest that SCAV‐3 is expressed and localized on lysosomes, and loss of *scav‐3* causes damage to lysosome membranes.

### The persistent cell corpses in *scav‐3(ok1286)* are internalized but not digested

**Fig. 5:**
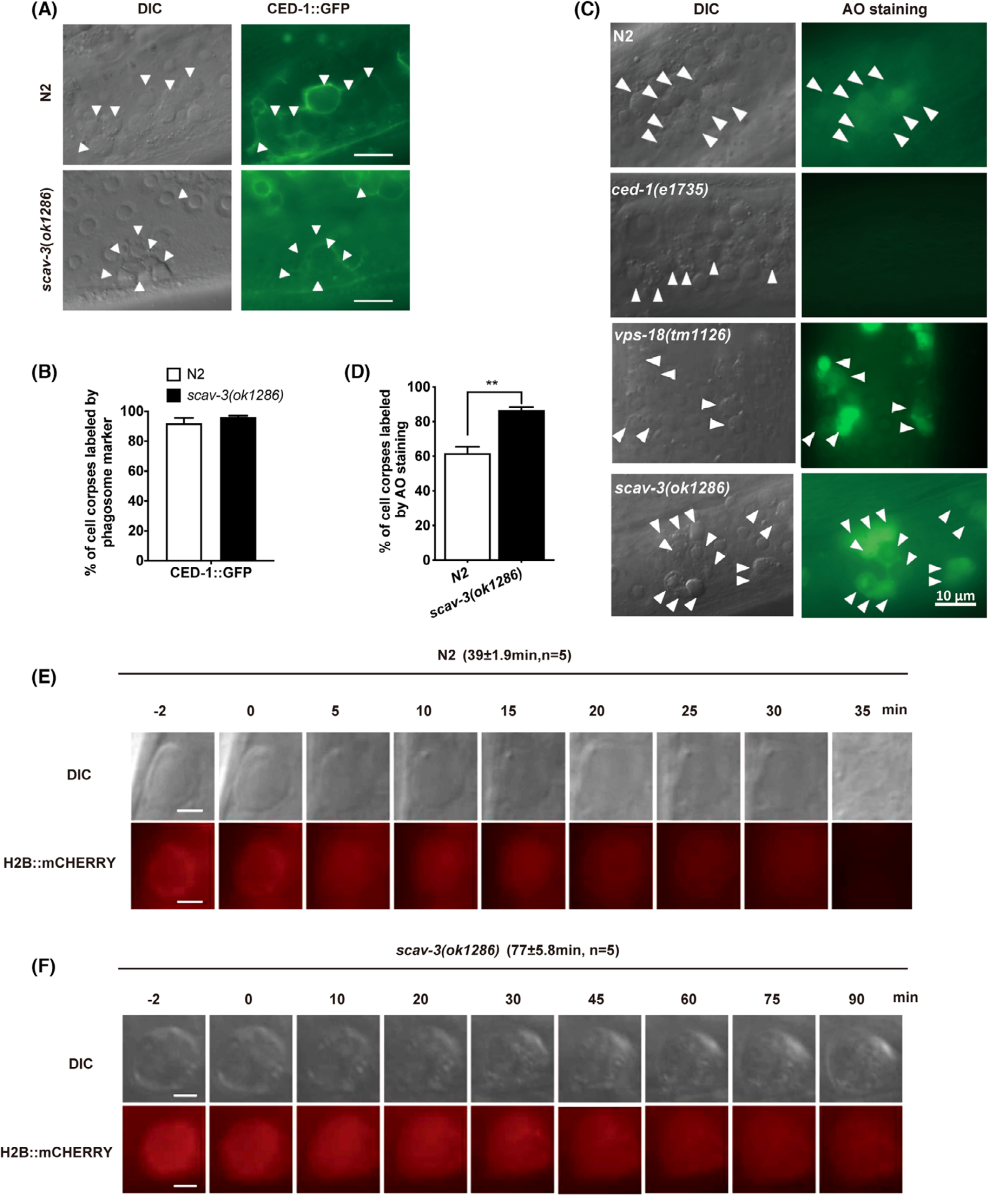
*scav‐3* is required for the degradation of cell corpses. (A) Representative images of cell corpse labeling by CED‐1::GFP, cell corpses indicated by white arrows; Bars, 10 μm. (B) Quantification (mean ± SEM) of the labeling of cell corpses by CED‐1::GFP. We analyzed ≥ 100 cell corpses from ≥ 20 animals for each strain, and data were derived from three biologically independent replicates. (C) AO staining of germ cell corpses in indicated animals. Arrows indicate cell corpses in N2, *ced‐1(e1735)*, *vps‐18(tm1125)*, and *scav‐3(ok1286)* worms. Bars, 10 μm. (D) Quantification (mean ± SEM) of AO‐positive germ cell corpses in wild‐type and indicated mutants. We analyzed ≥ 100 cell corpses from ≥ 20 animals for each strain, and data were derived from three biologically independent replicates. Time‐lapse recording of chromatin degradation of the germ cell corpses of both (E) N2 and (F) *scav‐3(ok1286)* animals. The numbers in parentheses indicate the average duration of cell corpses (mean ± SEM). Bars, 2 μm. Data were analyzed with the unpaired *t*‐test. **P < 0.01.

To investigate how *scav‐3* influences the clearance of cell corpses, we first examined whether *scav‐3* affected the recognition of ACs by phagocytes. In *scav‐3(ok1286)* mutant animals, the majority of the persisting germ cell corpses were surrounded by CED‐1::GFP generated by germ line sheath cells, indicating that *scav‐3* does not affect the recruitment of phagocytes around germ cell corpses (Fig. 5A,B). AO can be used to label the cell corpses internalized in live cells. We next used AO staining to determine whether the persisting cell corpses in *scav‐3(ok1286)* animals were internalized. In this experiment, the mutant *ced‐1(e1735)* served as a negative control because majority of cell corpses were not phagocytosed in this mutant, whereas the mutant *vps‐18(tm1125)* served as a positive control because most cell corpses were phagocytosed [27] but not degraded in this mutant. In the germ line of wild‐type animals, cell corpses were rapidly engulfed, of which 60% were AO positive. In contrast, only a small number of germ cell corpses in *ced‐1(e1735)* animals were labeled by AO, but majority of germ cell corpses in *vps‐18(tm1125)* animals were stained by AO. We found that the gonad ACs of *scav‐3(ok1286)* were rapidly engulfed by phagocytes, and the ratio of AO staining was higher than that of wild‐type *N2* (*P* < 0.05) (Fig. 5C,D). These results indicate that these persistent germ cell corpses in *scav‐3(ok1286)* were indeed internalized by the engulfing cells. To directly monitor the degradation process of cell corpses, we introduced in *scav‐3(ok1286)* mutants a transgenic marker H2B::mCHERRY [28], which is specifically expressed in the germ line to label chromatin. Our results showed that wild‐type and mutant animals had obvious ‘button‐like’ ACs at 0 min, and the H2B::mCHERRY fusion protein surrounded the cells as the starting point for degradation. In the wild‐type, the chromatin in early germ cell corpses was condensed and disappeared within 40 min (39 ± 1.9 min; *n* = 5). The chromatin in early germ cell corpses was also condensed in *scav‐3(ok1286)* mutants, it became diffusive in the later stage, and the mCHERRY signal remained for 80 min (77 ± 5.8 min, *n* = 5) (Fig. 5E,F), showing that chromatin breakdown is greatly delayed. These results reveal that the accumulation of cell corpses produced by the scav‐3 mutation is due to deficiencies in the digestion of cell corpses in engulfing cells.

### SCAV‐3 affects lysosome digestion activity

**Fig. 6:**
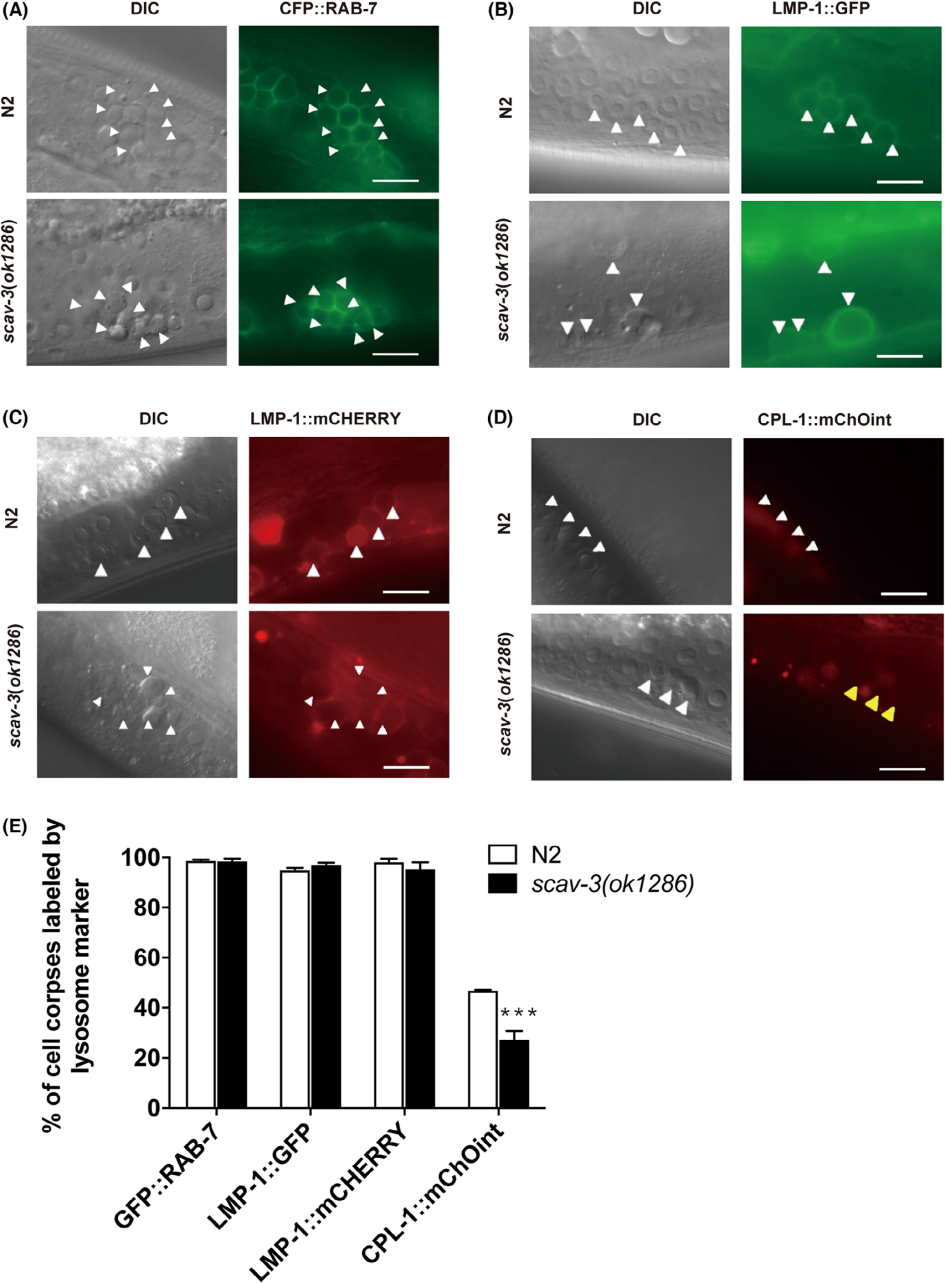
Characterization of phagosome maturation process in *scav‐3* mutants. Representative images of cell corpse labeling by (A) GFP::RAB‐7, (B) LMP‐1::GFP, (C) LMP‐1::mCHERRY, and (D) CPL‐1::mChOint. Cell corpses indicated by white arrows; cell corpses unlabeled indicated by yellow arrows. Bars, 10 μm. (E) Quantification (mean ± SEM) of the labeling of cell corpses by different phagosomal markers shown in A–D. We analyzed ≥ 100 cell corpses from ≥ 20 animals for each marker, and data were derived from three biologically independent replicates. Comparisons were performed between N2 and *scav‐3(ok1286)* mutants for each marker using an unpaired *t‐*test. ***P < 0.001.

To clarify how SCAV‐3 affects AC degradation, we analyzed the recruitment of maturation‐required phagosomal components. We introduced integrated arrays expressing GFP‐fluorescent protein‐tagged phagosome markers (GFP::RAB‐7) [20, 29], a small GTPase required for late endosome–phagosome fusion, into *scav‐3(ok1286)* mutants. We found that phagosomes in *scav‐3(ok1286)* germlines are positive for GFP::RAB‐7 at a similar level as in the wild‐type, indicating that loss of *scav‐3* did not affect the phagosome maturation process (Fig. 6A,E). After phagosomes mature, they combine with lysosomes to form phagolysosomes. Then hydrolytic enzymes in lysosomes enter the phagosomes for degradation. Thus, SCAV‐3 should function at the stage of cell corpses digestion in phagolysosomes. To examine whether the internalized germ cell corpses (phagosomes) fused with lysosomes in both wild‐type and *scav‐3* mutants, we introduced integrated arrays expressing GFP or mCherry fluorescent protein‐tagged lysosome markers into *scav‐3(ok1286)* mutants, including LMP‐1(LMP‐1::mCHERRY and LMP‐1::GFP), a lysosomal membrane protein [30]. We discovered that phagosomes in *scav‐3(ok1286)* germ lines exhibit similar levels of LMP‐1 positivity as those in wild‐type (Fig. 6B,C,E). To examine whether hydrolytic enzymes in lysosomes entering the phagosomes were affected in *scav‐3(ok1286)*, we introduced integrated arrays expressing mChOint fluorescent protein‐tagged lysosome hydrolytic enzymes, CPL‐1(CPL‐1::mChOint), into *scav‐3(ok1286)* mutants [31]. We found that phagosome association with CPL‐1::mChOint was significantly reduced in *scav‐3(ok1286)* than in wild‐type (Fig. 6D,E), indicating that loss of *scav‐3* affected the hydrolytic enzyme CPL‐1 in lysosomes entering the phagosomes. Thus, in *scav‐3(ok1286)* mutants, although LMP‐1‐positive organelles, likely aberrant lysosomes, could be recruited to the cell corpse‐containing phagosome and were shown to be able to fuse further into a phagolysosome, hydrolytic enzymes leaked from the damaged lysosome. Thus, the digestive function of lysosomes was impaired, resulting in the accumulation of undigested cell corpses. These results indicate that the loss of function of SCAV‐3 affects lysosome digestion activity during the AC clearance process, resulting in the accumulation of ACs.

## Discussion

There are three scavenger receptor class B members in vertebrates: CD36, scavenger receptor SR‐BI, and lysosomal integral membrane protein‐2 (LIMP‐2, also known as SCARB2 or CD36B like‐2). CD36 and SR‐BI have been implicated in the engulfment of ACs in mammals [16]. SCAV‐1–6 consists of the six homologs of class B scavenger receptors in *C. elegans*. However, whether they affect apoptosis is not clear. Here, we found that only genetic inactivation of *scav‐3* gave rise to a substantial increase of both embryonic and germ cell corpses. The SCAV family appeared not to be functionally redundant during AC clearance, as only SCAV‐3 affects the clearance of ACs. However, SCAV‐3 and other phagocytic receptors may together maintain the phagocytosis of ACs. The scavenger receptors CD36 and SR‐BI are highly expressed in macrophages and are able to bind anionic phospholipids, serine phospholipids, and ACs, which is one of the important conditions for macrophages to participate in AC clearance [17]. In this paper, we found that the lysosomal membrane protein SCAV‐3 involved in the clearance process of ACs, unlike mammals, SCAV‐3 seems not to be involved in the binding process, but to degrade ACs by maintaining the membrane integrity of lysosomes. Our further analysis revealed that defects in cell corpse clearance, not excessive apoptosis, were responsible for the increased cell corpses and that *scav‐3* functions in engulfing cells for cell corpse clearance. These findings established that SCAV‐3 plays a critical role in the clearance of ACs.

SCAV‐3 is the *C. elegans* homolog of human LIMP‐2, which is a type III glycoprotein in the membranes of lysosomes and endosomes. LIMP‐2 plays a vital role in the delivery of β‐glucocerebrosidase from the endoplasmic reticulum to lysosomes and has been identified as the cellular receptor for multiple virus entry into host cells [32]. Recent studies found that SCAV‐3 maintains lysosome integrity and dynamics in *C. elegans*, although lysosomal localization of β‐glucocerebrosidase is not affected by the loss of *scav‐3* [21]. Consistent with this study, we found that SCAV‐3 is widely distributed and localized on lysosomes, and loss of *scav‐3* causes damage to lysosome membranes.

**Fig. 7:**
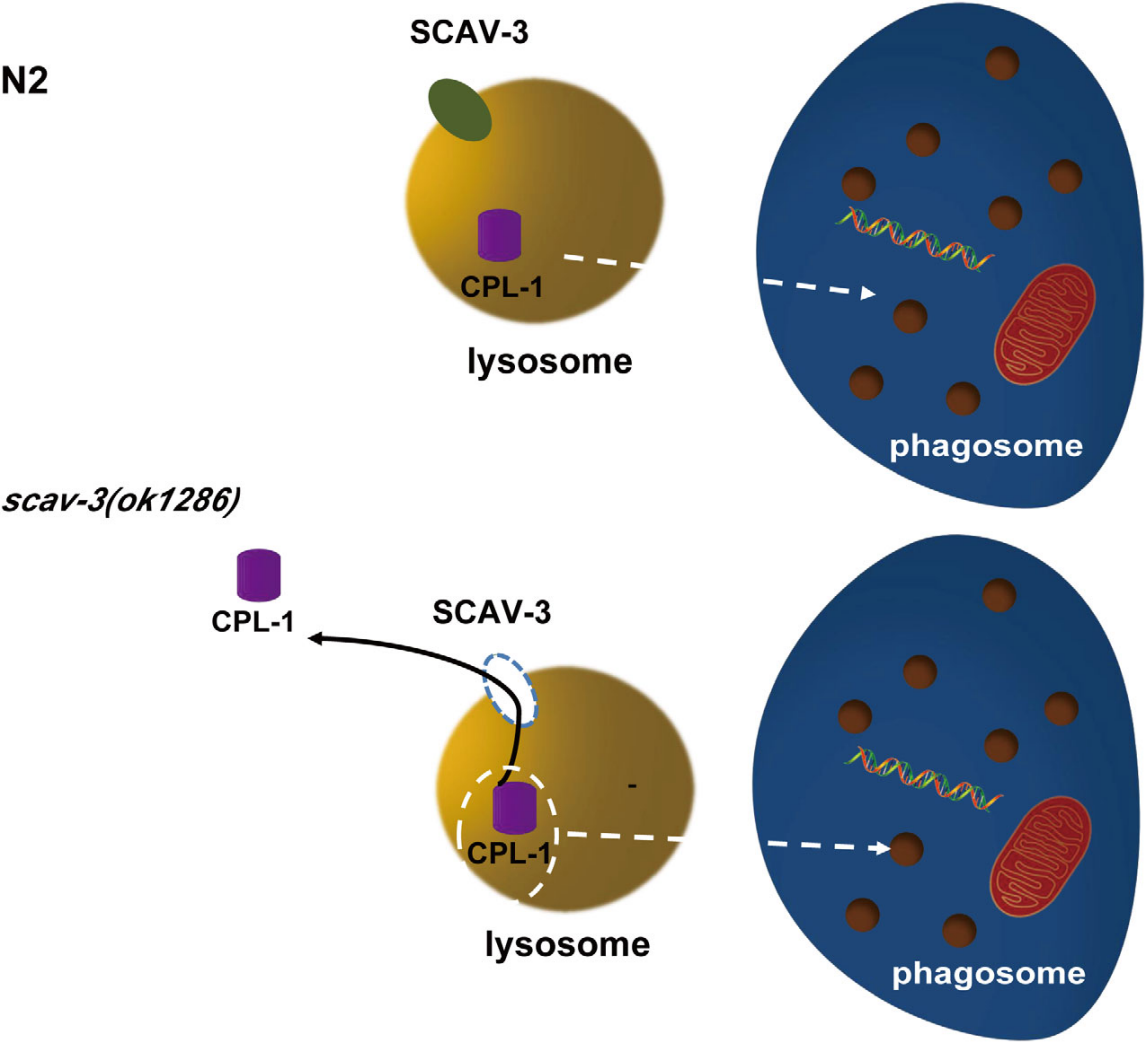
Schematic summary of the role of SCAV‐3 in AC clearance. Lack of SCAV‐3 function caused the integrity of the lysosomal membrane destroyed in *scav‐3(ok1286)*. Some hydrolases in the lysosome, such as CPL‐1, were leaked from the damaged lysosome, which affected lysosome digestion activity, leading to the accumulation of ACs.

In *C. elegans*, two parallel and partially redundant regulatory pathways induce cytoskeletal rearrangement of engulfing cells through recognition and transduction of engulfment signals, leading to pseudopod encapsulation for the internalization of ACs and finally to sealing to form phagosomes. Then, phagosomes fuse with lysosomes to form phagolysosomes [33]. Finally, the cell corpses are digested in the phagolysosome by lysosomal acid hydrolases, such as CPL‐1 and NUC‐1 [25, 33, 34]. We found that the persistent cell corpses in *scav‐3* mutants were internalized but failed to be degraded promptly. Our further analysis found that loss of *scav‐3* did not affect the phagosome maturation process or the recruitment of lysosomes to the cell corpse‐containing phagosome for further fusing into phagolysosome. In addition, we found lysosomal hydrolytic enzyme CPL‐1 could enter phagosomes but were reduced in phagosomes in *scav‐3* mutants (Fig. 7).

Lysosomes are ‘digestive factories’ in cells that can degrade extra‐ and intracellular material delivered by endocytosis, phagocytosis, and autophagy. Consequently, the dysfunction of lysosomes contributes to the pathogenesis of many disorders, such as lysosomal storage diseases, neurodegenerative disorders, and cancer [35, 36, 37]. Lysosomes contain a variety of hydrolytic enzymes that act in coordination to degrade all types of macromolecules [36]. Thus, the integrity of the lysosomal membrane becomes crucial, and it serves as the mechanical barrier that encloses potent luminal hydrolases and protects other cellular constituents from unwanted degradation [38]. Lack of SCAV‐3 function causes the integrity of the lysosomal membrane to be destroyed. Therefore, we propose that various hydrolases lysosomal hydrolases could still enter the phagosome during phagolysosome formation in *scav‐3* mutants, but that they are leaking from the damaged lysosome, thereby affecting lysosomal digestion activity and causing the accumulation of ACs.

## Conclusion

In this study, we investigate whether six homologs of scavenger receptors of class B in *C. elegans* affect apoptosis. We found that only loss‐of‐function *scav‐3* resulted in a large accumulation of cell corpses due to a failure in degradation rather than engulfment. SCAV‐3 is widely distributed and localized on lysosomes to maintain the integrity of the lysosomal membrane. Our further analysis indicates that in the process of phagolysosome formation in *scav‐3* mutants, hydrolases may be leaked from the damaged lysosome, leading to defects in the degradation of cell corpses.

## Conflict of interest

The authors declare no conflict of interest.

## Author contributions

HX conceived the study. AM, QF, and PL did most of the experiments. LY contributed to materials. HX and AM wrote the manuscript with feedback from all authors.

## Supporting information


**Fig. S1.** Knock down *scav‐3* caused accumulation of ACs in *C. elegans*. (A) Different stages of embryonic corpses were quantified (mean ± SEM) in the *scav‐3* RNAi‐treated. Fifteen embryos were scored at each stage for each strain. (B) The *scav‐3* RNAi‐treated germ cell corpses were quantified in different adult stages (h post L4). Fifteen adult worms were scored at each. Error bars represent SEM. Comparisons were performed with an unpaired t‐test. **p < 0.01, ***p < 0.001.

**Fig. S2.** *scav‐3(ok1286)* mutation affects ssGFP degradation. (A) Wild‐type animals expressing ssGFP controlled by a heat‐shock promoter were heat shocked for 60 min at 33°C, and the uptake and degradation of ssGFP in coelomocytes were monitored at indicated time points. Left, accumulation of ssGFP in body cavity, and the arrowheads indicate coelomocytes that are enlarged in the right pictures. Bars, 10 μm (left) and 10 μm (right). (B) *scav‐3(ok1286)* animals expressing ssGFP were treated and monitored as in described in A.

## Data Availability

The *C. elegans* strains and experimental data involved in the manuscript are available from Xiao's laboratory.
