# Supplementary figures and images for: SCAV‐3 affects apoptotic cell degradation in Caenorhabditis elegans
Source: FEBS Open Bio. 2023 Apr 2;13(5):867–80. doi: 10.1002/2211-5463.13599 (PMC10153301; doi:10.1002/2211-5463.13599)

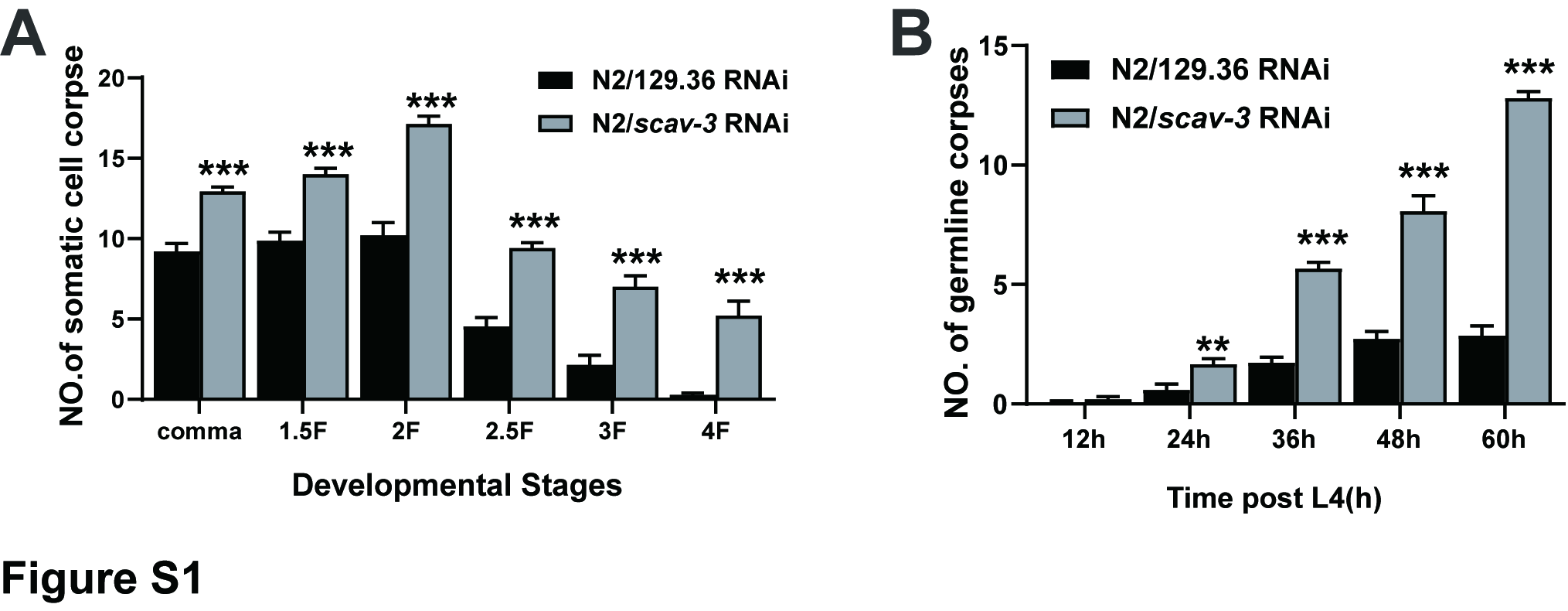

Supplement: Supplementary file 1 — Fig. S1. Knock down scav‐3 caused accumulation of ACs in C. elegans. (A) Different stages of embryonic corpses were quantified (mean ± SEM) in the scav‐3 RNAi‐treated. Fifteen embryos were scored at each stage for each strain. (B) The scav‐3 RNAi‐treated germ cell corpses were quantified in different adult stages (h post L4). Fifteen adult worms were scored at each. Error bars represent SEM. Comparisons were performed with an unpaired t‐test. **p < 0.01, ***p < 0.001. [file FEB4-13-867-s002.tif]

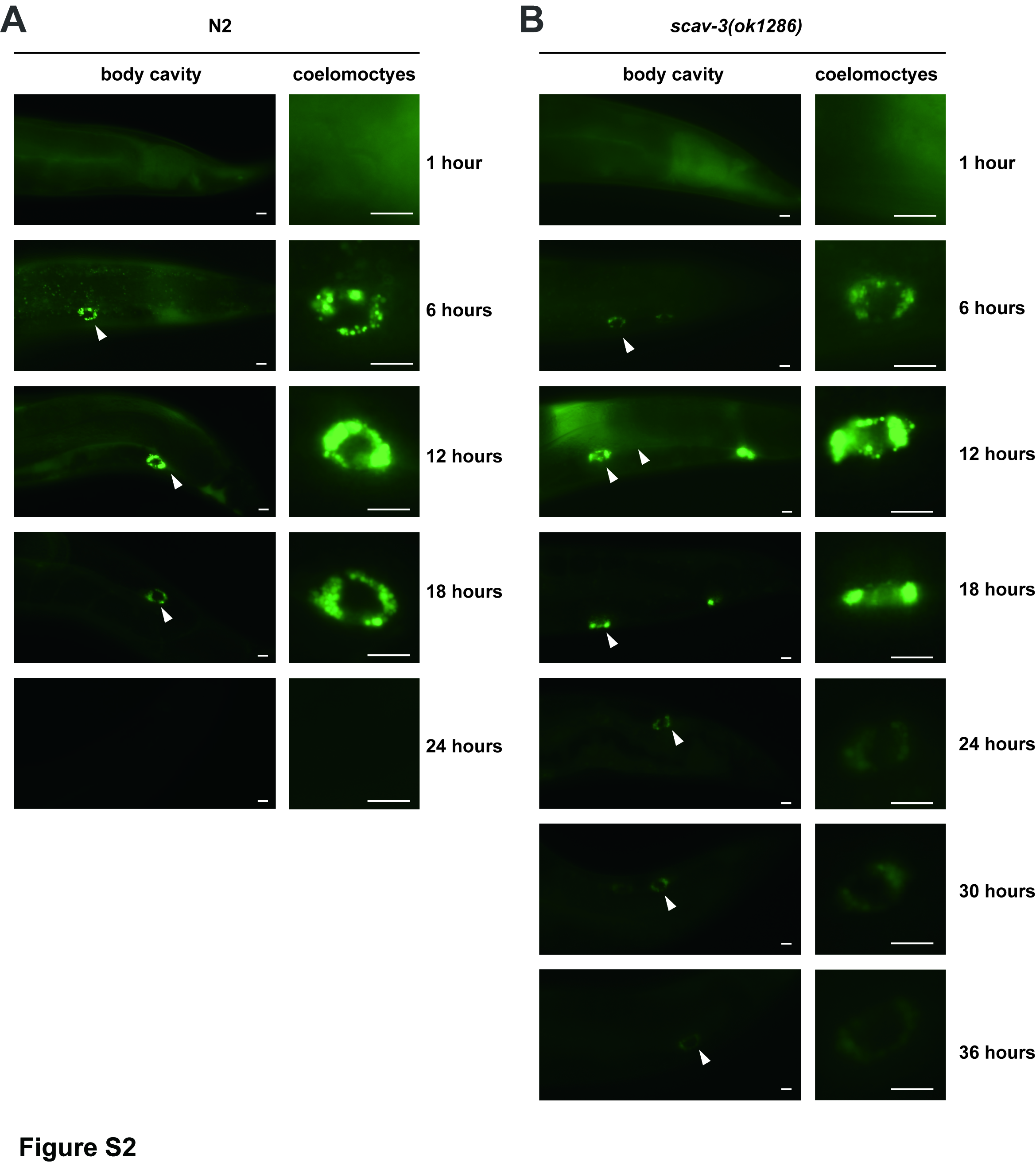

Supplement: Supplementary file 2 — Fig. S2. scav‐3(ok1286) mutation affects ssGFP degradation. (A) Wild‐type animals expressing ssGFP controlled by a heat‐shock promoter were heat shocked for 60 min at 33°C, and the uptake and degradation of ssGFP in coelomocytes were monitored at indicated time points. Left, accumulation of ssGFP in body cavity, and the arrowheads indicate coelomocytes that are enlarged in the right pictures. Bars, 10 μm (left) and 10 μm (right). (B) scav‐3(ok1286) animals expressing ssGFP were treated and monitored as in described in A. [file FEB4-13-867-s001.tif]
